# 

**Published:** 1904-12-24

**Authors:** 


### NOTICE TO CORRESPONDENTS.

All MSS., Letters, Books for Review, and other matters intended for the Editor should be addressed The Editor, "The Hospital," The Hospital Buildings, 28 & 29 Southampton Street, Strand, W.C.

The Editor cannot undertake to return rejected MS., even when accompanied by stamped directed envelope.

**Notice to Advertisers and Subscribers.**

All Advertisements, Orders for Copies of The Hospital, and Business Communications should be addressed to **THE MANAGER (Not to the Editor),** The Hospital, 28 & 29 Southampton Street, Strand, London, W.C.

The Cost of Subscription, post free, is as follows:—Per week, 3½d.; per quarter, 3s. 3d.; per half-year, 6s. 6d.; per annum, 13s. Foreign and Colonial:—Per annum, 19s. 6d., payable, in all cases, in advance.

**NOTICE.—Vol. XXXVI. of "The Hospital" (April to September 1904), in handsome cloth binding, is now on sale at the office of this Journal, price 10s. 6d. Cases for binding the Numbers contained in this Volume 2s. Index to Vol. XXXVI., 3½d., post free.**

**The Monthly part for December is now ready. Price 1s., by post, 1s. 3d.**

### BOOKS RECEIVED.

Baillière, Tindall and Cox.

"The Treatment of Syphilis." By Lieut.-Col. F. J. Lambkin.

John Bale, Son, and Danielsson, Limited.

Simple Medical Year Book.

The Pioneer Press, Allahabad.

"The Transvaal Burgher Camps, South Africa." By Lieut.-Col. S. J. Thomson, C.I.E.

The Theosophical Publishing Company.

"Vegetarian Savouries." By Mary Pope.

W. Blackwood and Sons.

"Practical Nursing." By Isla Stewart and H. E. Cuff, M.D (New edition, in one volume )

A. C. Fifield.

"The Day Boy and the Night Girl."

Willings, Limited.

"Willing's Press Guide, 1905."

Chatto and Windus.

"Fry's Royal Guide to the London Charities, 1905."

Rebman, Limited.

"Bright's Disease and Diabetes." By James Tyson, M.D.

### MEDICAL APPOINTMENTS.

J. P. Byrne, L.R.C.S., L.R.C.P.I., Senior Resident Surgeon, Provincial Hospital, Port Elizabeth, Cape Colony. D. L. Davies, M.D.Lond., Certifying Factory Surgeon for the Wisbech and Walsoken District of the Counties of Cambridge and Norfolk. Leigh Day, B.A., M.B., B.Ch.Oxon, M.R.C.S., L.R.C.P., Honorary Assistant Surgeon to the Essex and Colchester Hospital. K. Delany, L.R.C.P. and S.Irel., Certifying Factory Surgeon for the Carrick-on-Shannon District, County Leitrim. I. Malcolm Farquharson, M.B., F.R.C.P.Ed., Second Assistant Surgeon to the Ear, Nose, and Throat Department of the Royal Infirmary, Edinburgh. W. T. Hoyes, M.D.Canada, Clinical Assistant to the Chelsea Hospital for Women. Archibald Kidd, M.R.C.S., L.R.C.P., D.P.H., Medical Officer of Health, Port Elizabeth, South Africa. J. Rogers, L.R.C.P.andS.Irel., Certifying Factory Surgeon for the Ballyfarnon District, County Sligo. M. Tench, M.D.Brux, L.R.C.P., M.R.C.S., Certifying Factory Surgeon for the Dunmow District, County Essex. G. J. Williams, M.B., B.S.Durh., Medical Officer and Vaccinator, Eastern District and Haltwhistle Union and Workhouse, Certifying Factory Surgeon, Haltwhistle Division, vice G. W. Pickering, L.R.C.P., L.R.C.S., resigned.

**"The Hospital" Institutional Library.**

"Hospitals and Asylums of the World." 4 Vols., with a Portfolio of Plans, complete £12 12s.

"Burdett's Hospitals and Charities, 1904." 5s. net.

"Burdett's Official Nursing Directory." 8s. net.

"Cottage Hospitals, General, Fever, and Convalescent." 10s. 6d.

"Uniform System of Accounts for Hospitals and Public Institutions." New Edition. 4s. net.

"Hospital Expenditure: The Commissariat." 2s. 6d.

"A Legal Handbook for the Use of Hospital Authorities." 2s. 6d.

"The Cottage Hospital Case Book or Register of Patients." 10s. and 12s. 6d.

All these are published by the Scientific Press, Ltd., and may be obtained through any bookseller, or direct from the publishers, 28 and 29 Southampton Street, Strand, London, W.C.

### Medical Appointments.

NORTH STAFFORDSHIRE INFIRMARY AND EYE HOSPITAL, HARTSHILL, STOKE-UPON-TRENT.

WANTED, a HOUSE SURGEON. Salary £110 per annum, increasing by £10 per annum at the discretion of the Committee, with Furnished Apartments, Board and Washing.

Candidates must be duly registered and possess a diploma or degree in Surgery from the College of Surgeons in London, Edinburgh, or Dublin, or from one of the Universities; and a diploma, degree or license in Medicine from a University or duly recognised licensing body of Great Britain or Ireland.

Applications, giving age, with not more than six testimonials, to be sent to the Secretary and House Governor not later than the 3rd JANUARY, 1905.

The selected candidates will be required to attend before the General Committee on the day of Election, January 11th, of which due notice will be given.

Canvassing prohibited.

By order of the Committee,
ALBERT E. ROYCE,
December 12th, 1904. Secretary and House Governor.

N.B.—Cæteris Paribus, preference will be given to one who has previously held the post of House Surgeon in a General Hospital. (3212)

ST. PANCRAS, LONDON.

The Guardians of this Parish are prepared to receive applications for the joint appointments of MEDICAL SUPERINTENDENT of the South Infirmary, Cook's Terrace, Pancras Road, and Medical Officer of the Workhouse adjacent, with salary as Medical Superintendent of the South Infirmary at the rate of £250 per annum, rising by annual increments of £25 to £350 per annum, with furnished apartments, coals, and lighting but not domestic service; and as Medical Officer of the Workhouse at the rate of £100 per annum.

The gentleman appointed will be required to enter into a contract for Vaccination.

Candidates must be Registered Medical Practitioners, qualified by law to practise both Medicine and Surgery in England and Wales.

Application to be made on forms to be obtained at my office as below.

Selected candidates will be informed when and where they may attend a meeting of the Guardians.

Canvassing directly or indirectly will be deemed a disqualification.

ALFRED A. MILLWARD,
Clerk to the Guardians.

Town Hall, Pancras Road, N.W.
December 15th, 1904. (3422)

### IMPORTANT NOTICE.

A Useful Xmas Present for Nurses.

THE

### HOSPITAL

### Calendar & Notebook

FOR

### 1905.

A Useful Xmas Present for Nurses.

**This compact and useful little Notebook, which is of a convenient size for the pocket, is in appearance an exact reproduction of THE HOSPITAL Journal in miniature and contains much information of value to nurses including: Hints for nurses; How to advertise; How to reply to an advertisement; Hints on how to accept a post; Measures; Apothecaries' and other weights, &c., &c.**

### To Nurses.

THE HOSPITAL Calendar and Notebook will be forwarded **Post Free** on receipt of **Two Penny Stamps.** Nurses requiring additional Copies can obtain One Dozen, post free, for Nine Penny Stamps.

**BINDING CASES, in New Pegamoid Leather Cloth, gilt lettered, specially designed to take THE HOSPITAL Calendar and Notebook, with loop for hanging from Wallet or Chatelaine with One Copy of the Calendar, 3½d. post free. These Cases are strongly made, and can be used from year to year, as they do not contain the year nor any lettering other than the title of the Calendar.**

The Diary will shortly be ready, and it is advisable to make immediate application to avoid disappointment, as the edition is limited and no reprints will be issued when it is exhausted.

*Address*—The Manager, Calendar Dept., THE HOSPITAL,
28 & 29 Southampton Street, Strand, London, W.C.

